# Antiviral Treatment of HCV-Infected Patients with B-Cell Non-Hodgkin Lymphoma: ANRS HC-13 Lympho-C Study

**DOI:** 10.1371/journal.pone.0162965

**Published:** 2016-10-17

**Authors:** Laurent Alric, Caroline Besson, Nathanael Lapidus, Juliette Jeannel, Jean-Marie Michot, Patrice Cacoub, Danielle Canioni, Stanislas Pol, Frédéric Davi, Pascaline Rabiega, Loic Ysebaert, Delphine Bonnet, Olivier Hermine

**Affiliations:** 1 Department of Internal Medicine and Digestive Diseases, CHU Purpan, UMR 152, IRD Toulouse 3 University, Toulouse, France; 2 Université Paris sud Faculté de Médecine Kremlin Bicêtre, AP-HP Hôpital Bicêtre, Le Kremlin-Bicêtre, IMVA INSERM U1184, Paris, France; 3 Sorbonne Universités, UPMC Univ Paris 06, INSERM, Institut Pierre Louis d’épidémiologie et de Santé Publique (IPLESP UMRS 1136), Department of Public Health, Hôpital Saint-Antoine, AP-HP, Paris, France; 4 Paris 6 Pierre et Marie Curie University, UMR 7211, INSERM, UMR S 959, CNRS, UMR 7211, AP-HP, Department of Internal Medicine, Hôpital Pitié-Salpêtrière, Paris, France; 5 Paris 5 Descartes University, AP-HP, Department of Pathology, Hôpital Necker, Paris, France; 6 Université Descartes, Inserm U-318 Institut Pasteur, AP-HP, Department of hepatology, Hôpital Cochin, Paris, France; 7 Paris 6 Pierre et Marie Curie University, AP-HP, Department of Biological Hematology and Cytogenetic, Hôpital Pitié-Salpêtrière, Paris, France; 8 Department of Hematology IUCT oncopole, CHU, Toulouse, France; 9 Paris 5 Descartes University, AP-HP, INSERM U 1163, CNRS ERL 8254, Department of Adult Hematology and Imagine Institute, Hôpital Necker, Paris, France; Kaohsiung Medical University Chung Ho Memorial Hospital, TAIWAN

## Abstract

Hepatitis C virus (HCV) infection is associated with lymphoproliferative disorders and B-cell non-Hodgkin lymphomas (B-NHLs). Evaluation of the efficacy and safety profiles of different antiviral therapies in HCV patients with B-NHL is warranted. Methods: First, we evaluated the sustained virologic response (SVR) and safety of Peg-interferon-alpha (Peg-IFN) + ribavirin +/- first protease inhibitors (PI1s) therapy in 61 HCV patients with B-NHL enrolled in a nationwide observational survey compared with 94 matched HCV-infected controls without B-NHL. In a second series, interferon-free regimens using a newly optimal combination therapy with direct-acting antiviral drugs (DAAs) were evaluated in 10 patients with HCV and B-NHL. Results: The main lymphoma type was diffuse large B-cell lymphoma (38%) followed by marginal zone lymphoma (31%). In the multivariate analysis, patients with B-NHL treated by Peg-IFN-based therapy exhibited a greater SVR rate compared with controls, 50.8% vs 30.8%, respectively, p<0.01, odds ratio (OR) = 11.2 [2.3, 52.8]. B-NHL response was better (p = 0.02) in patients with SVR (69%) than in patients without SVR (31%). Premature discontinuation of Peg-IFN-based therapy was significantly more frequent in the B-NHL group (19.6%) compared with the control group (6.3%), p<0.02. Overall, survival was significantly enhanced in the controls than in the B-NHL group (hazard ratio = 34.4 [3.9, 304.2], p< 0.01). Using DAAs, SVR was achieved in 9/10 patients (90%). DAAs were both well tolerated and markedly efficient. Conclusions: The virologic response of HCV-associated B-NHL is high. Our study provides a comprehensive evaluation of different strategies for the antiviral treatment of B-NHL associated with HCV infection.

## Introduction

Hepatitis C virus (HCV) infection leads to chronic liver disease and is also associated with extra-hepatic manifestations [1], such as mixed cryoglobulinemia (MC) and B-cell non-Hodgkin lymphomas (B-NHLs) [2–4]. Numerous studies have demonstrated that HCV-infected individuals have an increased risk of developing B-NHL [2–4]. HCV-associated B-NHLs are more often marginal zone lymphomas (MZLs) and diffuse large B-cell lymphomas (DLBCLs) [2–4]. In addition, some specific B-NHLs associated with HCV infection, such as lymphoma with villous lymphocytes, have been described [5]. Although HCV infection is involved in B-NHL, the pathogenic mechanism of hematological injury remains unclear. However, a continuum between mixed cryoglobulinemia (MCs) and B-NHL has been demonstrated [2,3]. Clinical and pathological data are consistent with a stepwise model of lymphomagenesis induced by chronic antigenic stimulation that contributes to the occurrence of MC, MZL and transformed DLBCL [2–5].

Combination therapy with Peg-interferon-alpha (Peg-IFN) + ribavirin was the first demonstrably effective treatment for HCV infection. Later, the combination of Peg-IFN+ribavirin with the first protease inhibitors (PI1s) telaprevir or boceprevir was clearly shown to be more beneficial for HCV genotype 1-infected patients [6]. Currently, the second generation of direct-acting antiviral drugs (DAAs), such as sofosbuvir, ledipasvir, daclastavir and simeprevir, are associated with an increased sustained virologic response (SVR) in HCV infection and are the gold standard for treating HCV infection in Western countries [7].

Numerous studies have suggested that HCV treatment could improve cryoglobulinemia manifestations [1,8]. In patients with low-grade HCV-related B-NHL, HCV clearance is associated with B-NHL remission [9,10]. However, antiviral therapy of patients with B-NHL associated with HCV is not well defined and must be further evaluated. Indeed, published studies have been performed in a small number of patients using heterogeneous HCV therapies and revealed an association with a high rate of viral failure [5,9]. In addition, reliable data assessing the role of DAAs in the management of patients with B-NHL are not available.

Therefore, there is a critical need to assess the efficacy and safety profiles of different antiviral therapies in HCV patients with B-NHL and to evaluate the influence of virologic responses on B-NHL prognosis. The nationwide Lympho-C ANRS observational non-randomized survey assessed the SVR and safety profile of Peg-IFN + ribavirin +/- PI1s therapy in a cohort of HCV patients with B-NHL matched to HCV-infected controls without B-NHL. In a second prospective cohort, interferon-free regimens using a new optimal combination therapy with DAAs were evaluated in patients with HCV associated with B-NHL.

## Patients and Methods

### Patients

Twenty-six French centers enrolled adult patients with B-NHL and positive HCV viral load at lymphoma diagnosis. HCV infection was defined by the detection of anti-HCV antibodies by enzyme-linked immunosorbent assay (ELISA) with a confirmatory test including HCV RNA detection by standardized polymerase chain reaction (PCR). Patients with HIV or HBV co-infection were not included. Liver fibrosis was evaluated by a liver biopsy or by measuring the liver stiffness according to the manufacturer’s instructions (Fibroscan, Echosens, France). The results were expressed in kilopascals (kPa). Metavir F3 was defined by a liver stiffness of 9.5 to 12.4 kPa, and Metavir F4 cirrhotic patients were defined by values of up to 12.5 kPa.

Clinical and biological data for either lymphoma or HCV infection were collected once the lymphoma diagnosis and treatments were received. All events related to lymphoma or hepatitis C treatment and outcome were recorded during follow-up. MC was defined as greater than 0.05 g/L of serum cryocrit. For all patients, cytohistological data regarding B-NHL diagnosis were recorded. B-NHL diagnosis was established according to the World Health Organization (WHO) 2008 classification. Blood cytology, immunophenotyping and molecular analyses were performed in each center and revised by expert hematologists (DC, FD). Lymphoma staging was determined using the Ann Arbor system. Extra-nodal lymphoma was defined when at least one extra-nodal site was involved. The spleen was not considered an extra-nodal site. Bone marrow infiltration by tumor cells was considered extra-nodal involvement. Digestive tract and/or pancreatic involvement was considered digestive involvement.

Treatment for lymphoma, treatment response, relapse, living status, and cause of death were recorded. The choice of lymphoma treatment was made at the discretion of each local practitioner. Lymphoma management was recorded as chemotherapy +/- rituximab, rituximab alone, antiviral therapy alone, or combination antiviral therapy and chemotherapy. Lymphoma response to therapy was assessed using the revised 2007 Cheson criteria. These criteria were used to define complete response, partial response, progressive disease and relapse [11]. Overall survival (OS) was measured from lymphoma diagnosis and written informed consent to last follow-up or to death from any cause.

All of the patients provided written informed consent. Investigations were performed after approval by Ethics Committee (Necker Hospital, Paris CPP Ile de France II) and were approved by the French Department of Health and Human Services. The study was conducted according to the principles expressed in the Helsinki Declaration. The study is registered at ClinicalTrials.gov (Identification number NCT01545544).

### Study design and antiviral therapy

**Fig 1:**
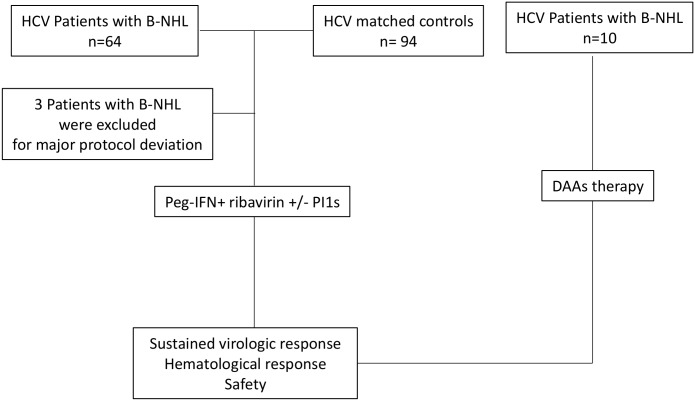
Flowchart. Peg-interferon-alpha (Peg-IFN), B-cell non-Hodgkin lymphoma (B-NHL) First generation of protease inhibitors (PI1s), Direct-acting antiviral drugs (DAAs).

The first cohort (Flowchart, Fig 1) started in November 2006 and was a part of a non-randomized multicenter nationwide observational survey (LYMPHO-C). We included 64 consecutive B-NHL patients treated for HCV infection. Among these 64 patients enrolled in the study, 3 patients were excluded for major protocol deviation: absence of antiviral therapy (n = 2) and lost to follow-up (n = 1). To evaluate SVR and the safety of antiviral therapy in this first cohort, the study population of 61 HCV patients with B-NHL was compared with a control group composed of 94 contemporary patients with chronic HCV infection without B-NHL from the Toulouse Purpan data bank as previously described [12,13]. Each HCV patient with B-NHL was matched with HCV controls without B-NHL for gender, HCV genotype, liver fibrosis severity according to Metavir F0-F1-F2 or F3-F4, IL28B genotype, and type of antiviral therapy. HCV genotype 2, 3, and 4-infected patients with B-NHL were matched with one control. Since the SVR was expected to be lower among subjects with HCV genotype 1 infection [6,7], we choose in this sub-group to increase the statistical power of the comparison between cases and controls. These most difficult to treat patients with HCV genotype 1 infection were matched with two patients from the control group. All patients received peg-IFNα2a (180 mg/week) + ribavirin (1000 to 1200 mg/day based on body weight). Some HCV genotype 1 patients were administered either 12 weeks of telaprevir (750 mg every 8 hours) combined with Peg-IFN+ ribavirin followed by 36 weeks of Peg-IFN + ribavirin or 4 weeks (lead-in phase) of Peg-IFN+ ribavirin followed by 44 weeks of Peg-IFN + ribavirin and boceprevir (800 mg every 8 hours, according to international label guidelines [6,7,14].

The second prospective cohort started in January 2014 and was composed of all consecutive HCV-infected patients with B-NHL treated with DAAs in the Toulouse-Purpan center (LA, DB, LY). Patients received 400 mg of sofosbuvir in combination with 90 mg of ledispavir or 60 mg of daclatasvir, 150 mg of simeprevir or 800–1200 mg of ribavirin once daily for 12 or 24 weeks according to the international label guidelines [7,14]. The choice of antiviral therapy combination was made at the discretion of each physician.

Quantification of the HCV RNA level was performed at baseline, again during antiviral therapy, and then 12 and 24 weeks following treatment withdrawal using real-time PCR (COBAS Amplicor/TaqMan, Roche Diagnostics, Basel, Switzerland) with a lower detection limit of 15 IU/ml. The response to antiviral therapy could be summarized as follows:

- rapid virological response (RVR), i.e., negative for HCV RNA after 4 weeks of therapy;
- sustained virological response (SVR), i.e., negative for HCV RNA 24 weeks after the end of treatment;
- relapse: patients who achieved undetectable HCV RNA levels at the end of antiviral therapy and then subsequently relapsed with positive HCV RNA after treatment withdrawal.

### Safety and adverse events

All of the patients were seen by their physicians at baseline, every 2 weeks during the first 2 months, every 4 weeks during the following phase of therapy, and then every 4, 12, and 24 weeks after treatment withdrawal. Adverse events were graded by investigators according to a modified WHO grading system. Non-life-threatening adverse events and hematological disorders were managed by reducing the ribavirin dose and/or giving erythropoietin (EPO) or granulocyte colony-stimulating factor (GCSF) at the discretion of the physician.

### Statistical analysis

Descriptive statistics included the mean (SD) or median with interquartile range (IQR), as appropriate, for continuous variables and frequency (%) for categorical variables. Conditional logistic regression models accounting for matching were used to compare baseline characteristics, SVR and premature discontinuation of antiviral therapy between B-NHL and controls. For SVR comparison, covariates associated with p<0.2 by univariate analysis or deemed clinically relevant were included in an Akaike information criterion (AIC)-based multivariate stepwise selection process. A maximum of four covariates was allowed in the tested models and p<0.05 was required for all of the covariates. The calibration of the final model was tested by the le Cessie–van Houwelingen goodness-of-fit test. Odds ratios (ORs) for the univariate analysis and the final multivariate model are provided. Kaplan-Meier estimator was plotted to compare OS between B-NHL and controls. A Cox regression model was used to estimate the univariate hazard ratio (HR) after assessing the proportional hazards assumption. Follow-up was defined from the B-NHL diagnosis to the last follow-up. A log transformation was used for HCV load, platelet and neutrophil counts and alanine aminotransferase (ALT) to better fit a normal distribution in the regression analyses (log10 for HCV load and log2 for all other covariates). All regression estimates are given with their 95% confidence intervals (CIs). Statistical significance was set at p<0.05 for all tests. All statistical analyses were performed using R software version 3.1 (R Foundation for Statistical Computing, Vienna, Austria).

## Results

### Characteristics of patients from the first cohort

As described above in flowchart (Fig 1), 61 patients with B-NHL matched with 94 controls were included in the study (Table 1). Criteria that were matched were gender, HCV genotype, IL28B genotype, severity of liver fibrosis, and antiviral therapy. Both groups were indistinguishable based on origin, body mass index (BMI), hemoglobin level, platelet count, neutrophil count, HCV load, ALT level and MC positivity. The patients with B-NHL were older than the patients in the control group (61.7 vs 53.9 years, p< 0.001). Of the HCV patients with B-NHL, 44.2% had an IL28B C/C genotype. Most of the patients with B-NHL were HCV genotype 1 (54%); however, the prevalence of HCV genotype 2 infection (24.5%) was high. According to the Metavir score, liver disease was moderate with a fibrosis score ≤ F2 in 55% of the patients. Most of the patients were treated with Peg-IFN + ribavirin therapy, and 10% were treated in combination with PI1s. Cytohistological data regarding lymphoma are presented in Table 1. The main type was indolent lymphoma (54%): MZL (31.1%), follicular lymphoma (18%) and mantle lymphoma (4.9%). However, 37.7% of the patients had aggressive DLBCL. Most of the patients exhibited extra-nodal lymphoma involvement (82%).

**Table 1:** Characteristics of the patients treated by Peg-IFN-based therapy.

	HCV patients with Non-Hodgkin’s lymphoma N = 61	HCV patients without Non-Hodgkin’s lymphoma N = 94	*P*
Age (years, IQR)	61.7 [50.4, 68.8]	53.9 [43.1, 61.8]	< 0.0001
Caucasians	48 (78.7%)	67 (71.3%)	0.06
BMI (kg/m2, IQR)	24.6 [21.3, 27.1]	24.7 [21.3, 28.6]	0.12
Haemoglobin level (g/dL):	13.1 [9.0, 16.9]	14.4 [11.5, 17.3]	< 0.02
Platelet count (G/L)	178.0 [31.6, 352.2]	200.0 [110.7, 359.8]	0.06
Neutrophil count (G/L)	3.1 [1.2, 10.1]	3.6 [1.4, 6.6]	0.144
Viral load (log10 (IU/ mL)	6.3 [4.6, 7.3]	6.1 [4.9, 7.8]	0.57
AST (IU/L)	36.0 [20.8, 508.4]	45.0 [24.5, 171.0]	0.40
ALT (IU/L)	31.5 [12.8, 603.8]	73.5 [30.9, 285.0]	0.05
Cryoglobulinemia	24 (25.3%)	11 (12.0%)	< 0.05
Male/Female	30/31	45/49	Matching criteria
HCV genotype (%)			Matching criteria
1	33 (54)	66 (70.2)	1: 2
2	15 (24.5)	15 (15.9)	1: 1
3	5 (8.1)	5 (5.3)	1: 1
4	6 (9.8)	6 (6.3)	1: 1
undetermined	2 (3.2)	2 (2.1)	1: 1
IL28B genotype (%)			Matching criteria
CC	27 (44.2)	33 (35.1)	
non CC; unknown	26 (42.6); 8 (13.1)	53 (56.3); 8 (8.5)	
Metavir fibrosis (%)			Matching criteria
F0F1F2	34 (55.7)	53 (56.3)	
F3F4	22 (36)	38 (40.4)	
Unknown	5 (8.1)	3 (3.1)	
Antiviral therapy (%)			Matching criteria
Peg-IFN+ ribavirin	54 (88.5)	82 (87.3)	
Peg-IFN+ ribavirin + first PI1s	7 (11.4)	12 (12.8)	
B-NHL histological groups:			
Diffuse large B-cell lymphoma	23 (37.7)	-	
Marginal zone lymphoma	19 (31.1)	-	
Follicular lymphoma	11 (18)	-	
Mantle lymphoma	3 (4.9)	-	
Other	5 (8.1)	-	
Lymphoma localization			
Nodal	11 (18)	-	
Extra nodal	50 (81.9)	-	

**Fig 2:**
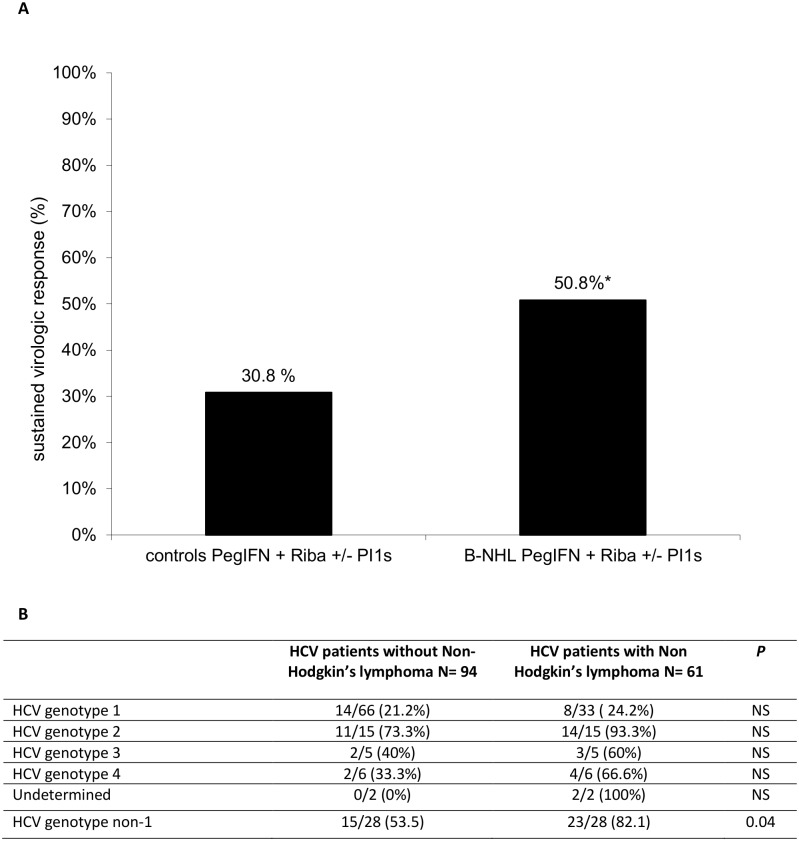
Sustained virologic response of the patients from the cohort treated by Peg-IFN-based therapy, * p< 0.02. First generation of protease inhibitors (PI1s). A: global sustained virologic response. B: sustained virologic response according to HCV genotype.

Virologic response response in the first cohort treated with Peg-IFN-based therapy:

The overall SVR rate (Fig 2A) was higher in the B-NHL group (50.8%) than in the control group (30.8%), p<0.02. SVR rate (Fig 2B) was not statistically different among subjects with HCV genotype 1 infection (24% in B-NHL group vs 21% in controls). In patients with HCV genotype non-1 infection (Fig 2B) SVR was higher in the B-NHL group (82.1%) than in the control group (53.5%), p = 0.04. The duration of antiviral therapy as well as the decrease of ribavirin or Peg-IFN dosage during therapy were similar in both groups and did not change the SVR rate. In Table 2, predictive factors of SVR are shown. SVR was higher for HCV genotypes 2 and 3 than for HCV genotypes 1 and 4, p<0.0001. Patients with the IL28B C/C genotype had a better SVR than patients with non C/C genotypes, p< 0.01. A lower platelet count at baseline decreased SVR, p = 0.05 (Table 2). The SVR rate was not significantly influenced by liver fibrosis or presence of MC. A higher cumulative dosage of ribavirin, EPO use or blood transfusion was not associated with an increase in SVR. Lymphoma chemotherapy was initiated after treatment of HCV in 19 patients (31.1%) and before antiviral therapy in 31 patients (50.8%). Eleven (18%) patients received antiviral therapy without chemotherapy for lymphoma. Antiviral therapy prior to chemotherapy for B-NHL was not associated with a better SVR. The logistic regression analysis (Table 2) demonstrated that only 5 factors were independently associated with SVR: patients with B-NHL, HCV genotypes 2 and 3, the IL28 C/C genotype, a higher platelet count at baseline and the use of PI1s in association with Peg-IFN+ribavirin. Multivariate analysis confirmed that patients with B-NHL had a better SVR than controls p<0.01, OR = 11.2 [2.3, 52.8].

**Table 2:** Predictive factors associated with sustained virologic response in the cohort treated by Peg-IFN-based therapy.

	Univariate analysis			Multivariate analysis		
	OR	CI 95%	*P*	OR	CI 95%	*P*
B-NHL vs controls	2.81	1.26–6,29	<0.02	11.2	2.3–52.8	P<0.01
Diffuse large B cell lymphoma vs marginal zone lymphoma	1,36	0.46 64.01	0.57			
Age at antiviral therapy	0.98	0.93–1.03	0.36			
Gender	0.52	0.26–1.02	0.06			
BMI	1,65	0.89–3.04	0.11			
Duration of HCV infection	1.16	1.0–1.35	0.69			
HCV genotype						
2–3 vs 1–4	6.8	2.88–16.06	<0.0001	16.7	3.3–83.7	<0.001
IL28B CC vs non CC	3.98	1.84 68.59	<0.001	10.08	2.4–41.1	P<0.01
METAVIR						
F0-F2 vs F3-F4	1.03	0.51–2.10	0.93			
Cryoglobulinemia	0.74	0.12,6	0.83			
HCV load at baseline	0.87	0.39–1,94	0.73			
Hb level at baseline	1.19	0.73–1.94	0.48			
Platelet count at baseline	4.77	1.01–22.5	0.05	2.76	1.1–6.6	<0.03
Neutrophil count at baseline	7.24	1.05–50,0	<0.05			
ALT at baseline	0.99	0.54–1.82	0.98			
Tritherapy vs bitherapy	2.8	0.97–8.12	0.06	14.80	2.6–82.5	<0.01
Antiviral therapy before B-NHL chemotherapy	0.69	0.12–3.78	0.67			
Ribavirin dosage at baseline	1	1.00–1,00	0.34			
No change in Ribavirin dosage	1.52	0.33–6.94	0.59			
No change in Peg-IFN dosage	0.2	0.02–1,87	0.16			
Cumulative ribavirin dosage	1.01	1.00–1,01	0.15			
Antiviral therapy duration	1.03	0.98–1,07	0.3			
EPO use	0.59	0.14–2,48	0.47			
GCSF use	0.46	0.04–5.24	0.53			
Blood transfusion	1.55	0.17–14.0	0.69			

### Impact of virologic response on hematological response in the first cohort treated with Peg-IFN-based therapy

**Fig 3:**
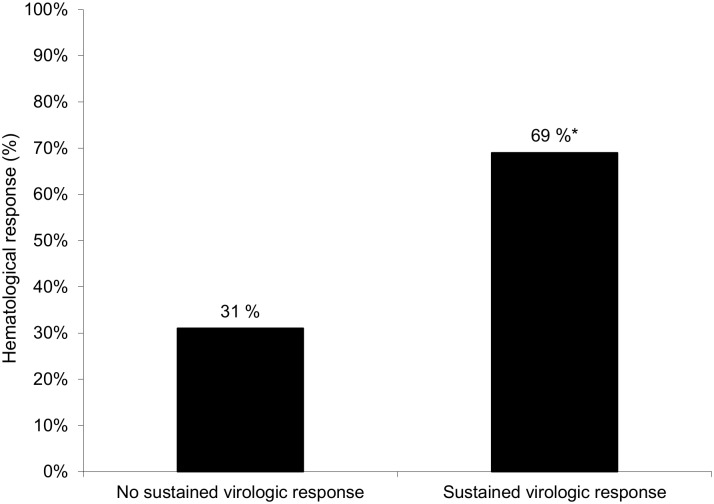
Haematological response of the patients from the B-NHL group treated by Peg-IFN-based therapy with or without sustained virologic response, * p = 0.02.

In the B-NHL group, haematological response was observed in 42/61 (68.8%) patients: 30 patients had a complete response and 12 a partial response. Conversely, 19 patients (31.1%) had a progression of lymphoma at the end of follow-up. The overall haematological response (Fig 3) was higher (p = 0.02) in patients with SVR (n = 29, 69%) than in patients without SVR (n = 13, 31%). Among the 42 patients with haematological response, 29 (69%) patients had SVR and only 2 of the 19 (10.5%) patients with lymphoma progression achieved a SVR, p<0.001, OR = 17.97 [3.4, 182.6]. Haematological response was not significantly different between patients with MZL or DLBCL. Haematological response was observed in 4 of 19 patients who had received antiviral therapy prior to chemotherapy for lymphoma.

### Safety and overall survival in the first cohort treated with Peg-IFN-based therapy

Premature discontinuation of antiviral therapy (Table 3) was significantly more frequent in the B-NHL group (19.6%) than in the control group (6.3%), p< 0.02. The use of GCSF was more frequent in the B-NHL group (13.3%) than in the controls (3.2%), p = 0.02. Infectious serious adverse events were more frequent in B-NHL patients (9.8%) than in controls (1%), p = 0.01. EPO use or blood transfusions did not differ between either group. The decrease of Peg-IFN or ribavirin dosage was similar in both groups. Only one patient with B-NHL died during antiviral therapy, and no patients in the control group died during antiviral therapy.

**Table 3:** Safety in the cohort treated by Peg-IFN-based therapy.

	HCV patients with Non-Hodgkin’s lymphoma	HCV patients without Non Hodgkin’s lymphoma	*P*
Premature discontinuation (%)	12/ 61 (19.6)	6/ 94 (6.3)	0.019
Decrease of Peg-IFN dosage (%)	12/60 (20)	14/ 90 (15.5)	0.5
Decrease of ribavirin dosage (%)	19/60 (31.6)	29/90 (32.2)	1
Blood transfusion (%)	8/ 62 (12.9)	4/ 94 (4.2)	0.06
EPO use (%)	20/ 61 (32.7)	19/ 93 (20.4)	0.09
GCSF use (%)	8/ 60 (13.3)	3/ 93 (3.2)	0.02
Blood adverse events grade 3/4(%)	12/ 60 (20)	29/ 90 (32.2)	0.13
Infectious adverse events grade3/4 (%)	6/ 61 (9.8)	1/ 90 (1)	0.01
Deaths at the end of follow-up (%)	6/ 61 (9.8)	1/94 (1)	0.01

**Fig 4:**
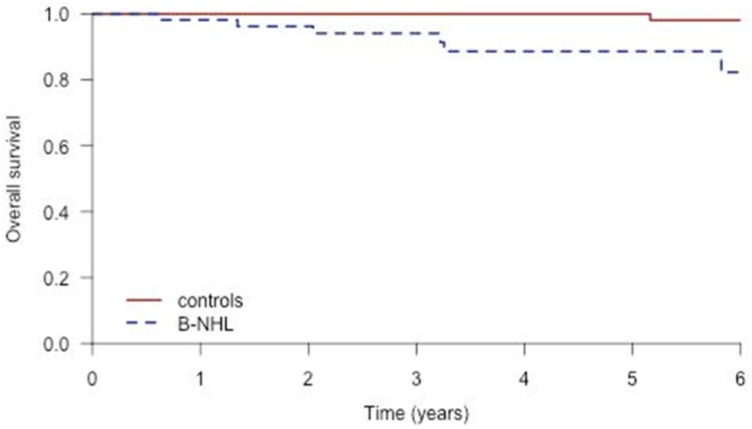
Overall survival of the patients with B-NHL and controls.

The time zero of survival was the day patients signed informed consent. After a follow-up of 6 years, we observed 6 deaths in the B-NHL group and one in the control group (Table 3). OS was significantly better (Fig 4) in the controls than in the B-NHL group (HR = 34.4 [3.9, 304.2], p< 0.01). Among the 6 patients from the B-NHL group, 3 deaths were related to severe infection; other causes of death were cardiovascular failure in 2 cases and lymphoma in one case. No deaths were related to antiviral therapy. In the control group, one death was related to hepatocellular carcinoma.

### Characteristics, treatment efficacy and safety in the second prospective cohort treated with IFN-free regimens with DAAs

Patient characteristics are detailed in Table 4. Ten consecutive patients with B-NHL prospectively treated with interferon-free regimens using DAAs were included in the cohort. The ages of the patients ranged from 52 to 77 years old, and the female gender ratio was 6/10. Patients had HCV infection with 6 different genotypes but HCV genotype 1 was the most prevalent, n = 6 (60%). Only one patient was cirrhotic, 6 were Metavir F3 and the 3 others had mild liver fibrosis. Seven of the 10 patients had MC. The main clinical features related to MC were purpura, peripheral neuropathy and glomerulonephritis. The main B-NHL cytopathological types were MZL (n = 6) and DLBCL (n = 3). Extra-nodal localization was observed in 7 patients, and most of the patients exhibited bone marrow involvement. Only one patient received only antiviral therapy without any lymphoma chemotherapy. For the 9 other patients, lymphoma chemotherapy was based on rituximab alone in 3 patients and rituximab in combination with other drugs in 6 patients. In all 9 of these cases, lymphoma chemotherapy and antiviral therapy were initiated together or within less than 4 weeks of each other. All of the patients were treated with a Peg-IFN-free regimen. Therapy with DDAs included sofosbuvir with ledipasvir (n = 4); simeprevir (n = 3); daclatasvir (n = 1) and ribavirin (n = 2). Rapid virologic response with negative HCV RNA at week 4 was observed in all of the patients. SVR was achieved in 9 of the 10 patients (90%). Only one HCV therapy failure was observed, despite a fair adherence to therapy, in a cirrhotic woman infected by HCV genotype 6 and treated for 24 weeks with sofosbuvir in combination with ledipasvir. HCV RNA was undetectable in this patient at week 4 and until week 12. After 14 weeks of antiviral therapy, HCV RNA was <15 UI/L but was quantifiable. A breakthrough occurred at week 16 and was confirmed at week 24. The NS5A-20V30A93T resistance-associated variant was detected in this patient.

**Table 4:** Characteristics and follow-up of the patients with HCV infection and B-NHL treated by direct-acting antiviral drugs (DAAs). Marginal zone lymphomas (MZLs); Diffuse large B-cell lymphomas (DLBCLs).

Patient	All patients n (%)	1	2	3	4	5	6	7	8	9	10
Age (years)	52–77	75	56	74	73	51	65	53	77	71	52
HCV genotype:											
1a	2 (20)	-	yes	-	-	-	-	yes	-	-	-
1b	4 (40)	yes		-	yes	-	yes	-	yes	-	-
2	1 (10)		-	-	-	yes	-	-	-	-	-
3	1 (10)	-	-	-	-	-	-	-	-	-	yes
5	1 (10)	-	-	yes	-	-	-	-	-	-	
6	1(10)	-	-		-	-	-	-	-	yes	-
Metavir fibrosis											
F0-F2	3 (30)	-	yes	yes	-	yes	-	-	-	-	-
F3-F4	7 (70)	yes		-	yes	-	yes	yes	yes	yes	yes
Cytohistopathological types:											
- MZL	6 (60)	-	-	yes	yes	yes	-	yes	-	yes	yes
- DLBCL	3 (30)	yes	-	-	-	-	yes	-	yes	-	
- other	1 (10)		yes	-	-	-	-	-	-	-	-
DAAs therapy:											
Sofosbuvir+ribavirin	2 (20)	yes	-	-	-	yes	yes	-	-	-	-
sofosbuvir+simeprevir	3 (30)		yes	yes	-	-	-	yes	-	-	-
sofosbuvir+daclatasvir	1 (10)	-		-	-	-	-	-	-	-	yes
sofosbuvir+ledipasvir	4 (40)	-	-	-	yes	-	-	-	yes	yes	yes
B-NHL therapy:											
none	1 (10)	-	-	-	-	-	-	-	-	yes	-
rituximab alone	3 (30)	-	-	yes	-	yes	-	yes	-	-	-
rituximab+polychemotherapy	6 (60)	yes	yes	-	Yes	-	yes	-	yes	-	yes
Sustained virological response	9 (90)	yes	yes	yes	Yes	yes	yes	yes	yes	-	yes
B-NHL response:											
complete response	9 (90)	yes	yes	yes	yes	yes	yes	yes	yes	-	yes
partial response	1 (10)			-	-	-	-	-	-	yes	
progression	0	-	-	-	-	-	-	-	-	-	-
SAEs grade ¾ related to DAAs	0	-	-	-	-	-	-	-	-	-	-

Nine of our 10 patients achieved a favorable hematological response with a post-treatment follow-up up to 6 months. The only patient who had a partial hematological response was the patient with virologic failure. The safety profile of therapy with DAAs was very good. In most of the patients, only minor side effects, such as fatigue, insomnia or pruritus, were observed as associated with treatment with DAAs. Only one patient, the cirrhotic woman who failed in response to sofosbuvir+ ledipasvir therapy, had a grade 3 adverse event related to liver failure with ascites that required hospitalization. No severe adverse event (SAE) 3/4 was observed for the other 9 patients (90%). During lymphoma polychemotherapy, 2 out of the 6 patients required EPO and 3 out of the 6 patients required GCSF.

## Discussion

HCV infection is associated with lymphoproliferative disorders [2–5]. Essential cryoglobulins are the predominant extra-hepatic manifestation of HCV infection [1,8]. Most cases of MC are associated with HCV infection [15,16]. Chronic stimulation of B lymphocytes by HCV most likely leads to the selection of a B clone producing monoclonal immunoglobulin component. Clinical studies have outlined a continuum from MC to low grade then aggressive lymphomas [2–4,17–19]. It was previously suggested that HCV antiviral therapy could induce hematological responses and improve survival in these patients [3,5,9]. In the context of dramatic changes to anti-HCV therapy, the data concerning the efficacy and safety of different antiviral therapies are lacking. Therefore, the Lympho-C ANRS study assessed the efficacy and safety profile of different antiviral therapies, including new therapies with DAAs, in HCV patients with B-NHL.

In our study, the most frequent cytopathological types of B-NHL associated with HCV were MZL (31%) and DLBCL (37%). Extra-nodal localization was observed in most of our patients (81%). These results are in accordance with studies of previously published cohorts from Europe [17]. The link between HCV infection and MC or lymphomagenesis is not completely understood. A strong association is observed in Southern Europe [17–19] particularly in Italy or France, whereas these HCV-associated extra-hepatic diseases are less frequent in Northern Europe [2] or Northern America [20], suggesting regulation by genetic or environmental factors. Various studies have demonstrated that antiviral therapy could improve the outcome of B-NHL associated with HCV infection [5,21,22]. However, data regarding the efficacy and safety of different antiviral therapies are lacking. In the nationwide cohort Lympho-C study, surprisingly, we observed a statistically better SVR in patients with B-NHL treated with Peg-IFN + ribavirin +/- PI1s than in controls. SVR was achieved in 50% of the patients with B-NHL despite negative predictive factors, such as genotype 1 or non-CC IL28B genotype. SVR in patients with genotype 1 infection was similar in the B-NHL group (24.2%) and in controls (21.2%). SVR rate in pooled patients with HCV genotype non-1 infection was significantly higher in the B-NHL group (82.1%) than in the control group (53.5%). The number of patients included in each HCV genotype subgroups was not sufficient to reach statistical difference. In the overall population including all HCV genotypes, statistical power was increased and SVR was significantly better in BNHL-group than in controls. The better SVR observed in B-NHL group was not related to HCV genotype 1 ratio matching. In these difficult to treat patients, Peg-IFN-based therapy was successfully used in routine clinical practice despite frequent treatment discontinuation. Hermine et *al*. [5] also observed a very high SVR (7/9 patients) using interferon in patients with a specific type of splenic lymphoma with villous lymphocytes. In a small Italian study of 25 patients [21], SVR was achieved in 8/25 (32%) patients. However, in both studies, some patients were treated with interferon alone. Moreover, none were treated with PI1s, and IL28B status was lacking. In a recent study, the MaSVE group [23] also reported a high rate of SVR in patients with symptomatic MC without B-NHL using Peg-IFN + ribavirin therapy. In our study, multivariate analysis identified the usual positive predictive factors associated with SVR; however, unexpectedly, B-NHL was another one. The reasons for this better response to antiviral therapy in patients with B-NHL compared with the control group, who were carefully matched for the main predictive factors of SVR, remain to be determined.

In our study, haematological response was improved by SVR. B-NHL outcome was better in patients with SVR (69%) than in patients without SVR (31%). Lymphoma progression was very high (89.4%) in patients who failed to have SVR. In previous studies, antiviral therapy improved the prognosis of B-NHL [3,21,22]. In a recent study [22] performed in patients with indolent B-NHL, Peg-IFN +/- ribavirin therapy improved OS and progression-free survival. However, none of the patients included in this study [22] had DLBCL, whereas 30% of the patients we treated in our study had aggressive lymphoma. Nevertheless, in our study Peg-IFN-based therapy was poorly tolerated. The majority of B-NHL-treated patients suffered from adverse effects related to Peg-IFN-based therapy. Hematological toxicity as well as infections were more frequent in the B-NHL group compared with the control group. Our patients with B-NHL were likely more fragile because half of the patients received chemotherapy for lymphoma before the Peg-IFN-based therapy making them more susceptible to serious adverse effects. After 6 years of follow-up of our cohort treated with Peg-IFN-based therapy, the OS in the B-NHL group was significantly reduced compared with that in the matched controls.

Our study provides analysis of the efficacy and safety of Peg-IFN + ribavirin +/- PI1s therapy in this uncommon population of patients with B-NHL. Currently, DAAs have completely changed the face of HCV therapy [7,14]. In the general population, interferon-free regimens with DAAs are associated with both excellent efficacy and safety. No reliable data are available on the use of DAAs in patients with B-NHL. Only single case reports have been published [24,25]. In a well-documented case of splenic MZL [24], a combination of oral faldaprevir, deleobuvir and ribavirin achieved SVR as well as clinical and hematological responses. This study reported only one case with a very short follow-up. Another case [25] of HCV-disseminated MZL exhibited a complete response to DAAs. In both of these case reports, patients had indolent B-NHL. In our prospective study, we treated 10 new patients with various DAAs in combination therapy without Peg-IFN. Three of our patients had aggressive DLBCL, and all HCV genotypes were represented. Of the 10 patients, 9 had an SVR. Half of our patients had a viral failure to Peg-IFN-based therapy prior to therapy with DAAs. Only one virologic failure was observed in a cirrhotic decompensated patient infected by HCV genotype 6. Given that this patient had a severe liver disease, ribavirin was not associated with DAAs or with chemotherapy for lymphoma. The virologic failure was not related to immunosuppressive drugs. She was the only patient to receive antiviral therapy without any lymphoma chemotherapy. The efficacy of DAAs for this uncommon HCV genotype 6 has not been completely assessed in the general population, and few patients have been included in pivotal studies [7,14]. During viral breakthrough, the NS5A-20V30A93T resistance-associated variant was detected. All of the patients except this woman were administered chemotherapy for lymphoma before therapy with DAAs. We treated 3 patients with aggressive DLBCL concomitantly with polychemotherapy for lymphoma, and we observed a very rapid virologic response. A hematological response was achieved in 9 of 10 patients, and only a partial response was observed in the patient with virologic failure. The safety of DAAs was excellent in all patients. The data from the second prospective cohort treated with DAAs demonstrated that a high rate of SVR and a hematological response could be achieved using DAAs in various types of B-NHL. Nevertheless, in HCV patients with B-NHL, a putative beneficial anti-proliferative effect of Peg-interferon on lymphoma cannot be excluded. Prospective trials with DAAs will be required to assess the long term benefits and the best strategies to treat B-NHL associated with HCV infection. Taking into account the safety and antiviral efficacy of DAAs compared with Peg-IFN-based therapy, we suggest that DAAs must be introduced early after B-NHL diagnosis even if chemotherapy is required for aggressive DLBCL.

The limitation of our first cohort treated with Peg-IFN-based therapy is related to the possible selection of patients who were not randomized and to the fact that we used different antiviral therapies. To limit survey bias, the study population was compared with a control group composed of contemporary HCV-infected patients without B-NHL matched for the main predictive factors associated with antiviral therapy response. In addition, in the second cohort receiving DAAs, all of the patients were included prospectively. The main strength of our study is that it provides a comprehensive evaluation of different strategies for the antiviral treatment of B-NHL associated with HCV infection.

In conclusion, if Peg-IFN-based therapy was the past and DAAs the future, the putative beneficial immunomodulatory and anti-proliferative effects of interferon cannot be definitively eliminated. We suggest that improvement of SVR and safety using DAAs will be associated with a survival improvement of these specific patients. Because DAAs are revolutionizing the treatment of HCV infection, the data presented in the current study could set the stage for further prospective trials in various types of B-NHL associated with HCV.

## Appendix

### Members of the National ANRS HC-13 Lympho-C study group

L Alric, C Besson, O Hermine, JM Michot, H Driss, P Rabiega, Y Barthe, AS Desgouilles, F Carrat, B Terrier, P Cacoub, F Davi, F Suarez, D Sibon, C Settegrana, F Nguyen-Khac, H Merle-Béral, D Canioni, T Lazure, F Berger, F Charlotte, T Lazure, T Molina, S Roulland, B Nadel, Y Taoufik, H Chavez, C Féray, S Pol, V Leblond, J Gabarre, C Thiéblemont, V Petrov-Sanchez, M Simony, JF Delfraissy, O Lambotte, B Deau Fischer, L Sutton, V Morel, C Haioun, J Dupuis, S Bologna, H Tilly, JL Harrousseau, M Lamy, M Attal, N Mounier, P Colombat, O Casasnovas, G Salles, A Delmer, E Gyan, and L Sanhes.

## Abbreviations

Peg-IFN: Peg-interferon-alpha
B-NHL: B-cell non-Hodgkin lymphoma
PI1s: First generation of protease inhibitors
DAAs: Direct-acting antiviral drugs
SVR: sustained virological response
MC: Mixed cryoglobulinemia
MZLs: Marginal zone lymphomas
DLBCLs: Diffuse large B-cell lymphomas
SAEs: Severe adverse effects
RVR: rapid virologic response
